# A PorX/PorY and σ^P^ Feedforward Regulatory Loop Controls Gene Expression Essential for Porphyromonas gingivalis Virulence

**DOI:** 10.1128/mSphere.00428-21

**Published:** 2021-05-28

**Authors:** Chizhou Jiang, Dezhi Yang, Tangsiyuan Hua, Zichun Hua, Wei Kong, Yixin Shi

**Affiliations:** a School of Life Sciences, Arizona State University, Tempe, Arizona, USA; b The State Key Laboratory of Pharmaceutical Biotechnology, School of Life Science and School of Stomatology, Nanjing University, Nanjing, Jiangsu, China; c The Biodesign Center for Immunotherapy, Vaccines and Virotherapy, Arizona State University, Tempe, Arizona, USA; University of Iowa

**Keywords:** PorX/PorY, *Porphyromonas gingivalis*, extracytoplasmic function sigma factor P, feedforward loop, *in vitro* transcription, transcription regulation, two-component system, type IX secretion system, virulence factors

## Abstract

The PorX/PorY two-component system in the periodontal pathogen Porphyromonas gingivalis controls the expression of the *por* genes, encoding a type IX secretion system, and the *sigP* gene, encoding sigma factor σ^P^. Previous results implied that PorX/PorY and σ^P^ formed a regulatory cascade because the PorX/PorY-activated σ^P^ enhanced the *por* genes, including *porT*, via binding to their promoters. We recently showed that PorX also binds to the *por* promoters, thus suggesting that an alternative mechanism is required for the PorX/PorY- and σ^P^-governed expression. Here, our *in vitro* assays show the PorX response regulator binds to the *sigP* promoter at a sequence shared with the *porT* promoter and enhances its transcription, mediated by a reconstituted P. gingivalis RNA polymerase holoenzyme. Merely producing σ^P^ in *trans* fails to reverse the *porT* transcription in a *porX* mutant, which further argues against the action of the proposed regulatory cascade. An *in vitro* transcription assay using a reconstituted RNA polymerase-σ^P^ holoenzyme verifies the direct role of PorX in *porT* transcription, since transcription is enhanced by a pure PorX protein. Accordingly, we propose that the PorX/PorY system coordinates with σ^P^ to construct a coherent regulatory mechanism, known as the feedforward loop. Specifically, PorX will not only bind to the *sigP* promoter to stimulate the expression of σ^P^, but also bind to the *porT* promoter to facilitate the RNA polymerase-σ^P^-dependent transcription. Importantly, mutations at the *porX* and *sigP* genes attenuate bacterial virulence in a mouse model, demonstrating that this regulatory mechanism is essential for P. gingivalis pathogenesis.

**IMPORTANCE** The anaerobic bacterium Porphyromonas gingivalis is not only the major etiologic agent for chronic periodontitis, but also prevalent in some common noncommunicable diseases such as cardiovascular disease, Alzheimer's disease, and rheumatoid arthritis. We present genetic, biochemical, and biological results to demonstrate that the PorX/PorY two-component system and sigma factor σ^P^ build a specific regulatory network to coordinately control transcription of the genes encoding the type IX secretion system, and perhaps also other virulence factors. Results in this study verify that the response regulator PorX stimulates the expression of the genes encoding both σ^P^ and the type IX secretion system by binding to their promoters. This study also provides evidence that σ^P^, like the PorX/PorY system, contributes to P. gingivalis virulence in a mouse model.

## INTRODUCTION

The Gram-negative anaerobic bacterium Porphyromonas gingivalis is the major etiologic agent for chronic periodontitis. This pathogenic bacterium produces a repertoire of virulence factors, including specific cysteine proteases, also known as gingipains (1–3). Secretion of gingipains is mediated by a type IX secretion system (T9SS) in a manner dependent on the *porX* and *porY* gene products, PorX and PorY, which were proposed to form a two-component regulatory system (TCS) by the Nakayama laboratory (4). Particularly, their results showed that PorX, the response regulator, and PorY, the histidine kinase, were able to upregulate the expression of the T9SS-encoding genes (referred to as the *por* genes, herein) including *porT*, *sov*, *porP*, *porK*, *porL*, *porM*, and *porN* (4). Furthermore, Kadowaki et al. carried out a surface plasmon resonance analysis and showed that PorY could directly interact with, and subsequently phosphorylate, PorX (5), thus experimentally demonstrating that these two proteins should be the TCS cognate pair. However, it remains elusive whether the PorX/PorY system controls transcriptional regulation of the *por* genes directly and, if that is the case, how the response regulator PorX interacts with these target genes.

In accordance with the observations from Kadowaki et al. (5), the Vincent and Cascales laboratory used a bacterial two-hybrid system and confirmed the *in vivo* interaction between the PorX and PorY proteins (6). In contrast, their results suggested that PorX should be involved in the dynamics of the T9SS system via an interaction with the cytoplasmic domain of the T9SS component PorL (6). They further argued that PorX/PorY could not regulate the *por* genes since they failed to observe PorX binding to the *por* promoters in a P. gingivalis promoter/PorX reconstitution assay performed in Escherichia coli, in which PorX was heterologously expressed for testing its role in stimulating a plasmid-borne *gfp* (green fluorescent protein) gene controlled by a *por* promoter (6). However, we realized that the α, β, and β’ subunits of P. gingivalis RNA polymerase, which are encoded by the *PGN_1841*, *PGN_1571*, and *PGN_1570* genes, respectively, merely share 38%, 46%, and 50% of identity to the corresponding subunits of the E. coli RNA polymerase. Additionally, the major sigma factor σ^D^ (encoded by the *PGN_0638* gene) of P. gingivalis, which is a 287-residue protein, shares just 39% identity with the C-terminal 267-aa sequence of the E. coli housekeeping sigma factor σ^70^ (613 aa). Therefore, it is reasonable to postulate that P. gingivalis promoters cannot be recognized by the E. coli RNA polymerase-σ^70^ holoenzyme. Accordingly, the *gfp* expression would very unlikely be enhanced in the P. gingivalis promoter/PorX reconstitution assay conducted by Vincent et al. (6), even if these P. gingivalis promoters could bind the heterologously expressed PorX protein in E. coli. Recently, our study confirmed that the PorX/PorY system exerted a regulatory effect on the transcription of its target genes (7). The PorX/PorY regulatory role was further verified by our electrophoretic mobility shift assay (EMSA) and DNase footprinting analyses, which provided evidence that a PorX protein was able to bind the promoter of a *por* gene, *porT*, by interacting with two DNA sequences, i.e., site I (5′-tattacttccataattattgttgtg-3′) and site II (5′-gattcgcgcaaaaatacaatatcttt-3′) (7).

According to the observations from Kadowaki et al. (5), the PorX/PorY system upregulates transcription of the *sigP* gene (i.e., *PGN_0274*) that encodes an extracytoplasmic function sigma factor, σ^P^, and then σ^P^ mediates transcriptional activation of the *por* genes by binding to their promoters. In addition, their results suggest the function of σ^P^ is directly associated with PorX because these two proteins could be coimmunoprecipitated from P. gingivalis cell lysates (5). Based on these results, it seems reasonable to propose a regulatory cascade in which the PorX/PorY system stimulates σ^P^ and, in turn, σ^P^ enhances the *por* genes. However, it remains largely unknown whether the PorX/PorY system can directly or indirectly upregulate *sigP* transcription, and whether the PorX/PorY system and σ^P^ activate the *por* genes in a manner dependent on the regulatory cascade. In this study, we show that the PorX response regulator not only binds to the *sigP* promoter to activate transcription of this sigma factor gene, but also binds to a *por* promoter with a σ^P^-RNA polymerase holoenzyme to initiate its transcription. Based on our observations from both *in vitro* and *in vivo* analyses, we propose a feedforward regulatory loop to illustrate gene regulation in a manner dependent on the PorX/PorY system and σ^P^, which also provides an example to elucidate the coordinate interaction between two-component systems and their regulated sigma factors in gene regulation of P. gingivalis. Additionally, our results demonstrate that both the PorX/PorY system and σ^P^ are virulence factors that govern transcription of the genetic loci required for P. gingivalis virulence.

## RESULTS AND DISCUSSION

### PorX/PorY system and sigma factor σ^P^ coordinately regulate transcription in P. gingivalis.

**FIG 1:**
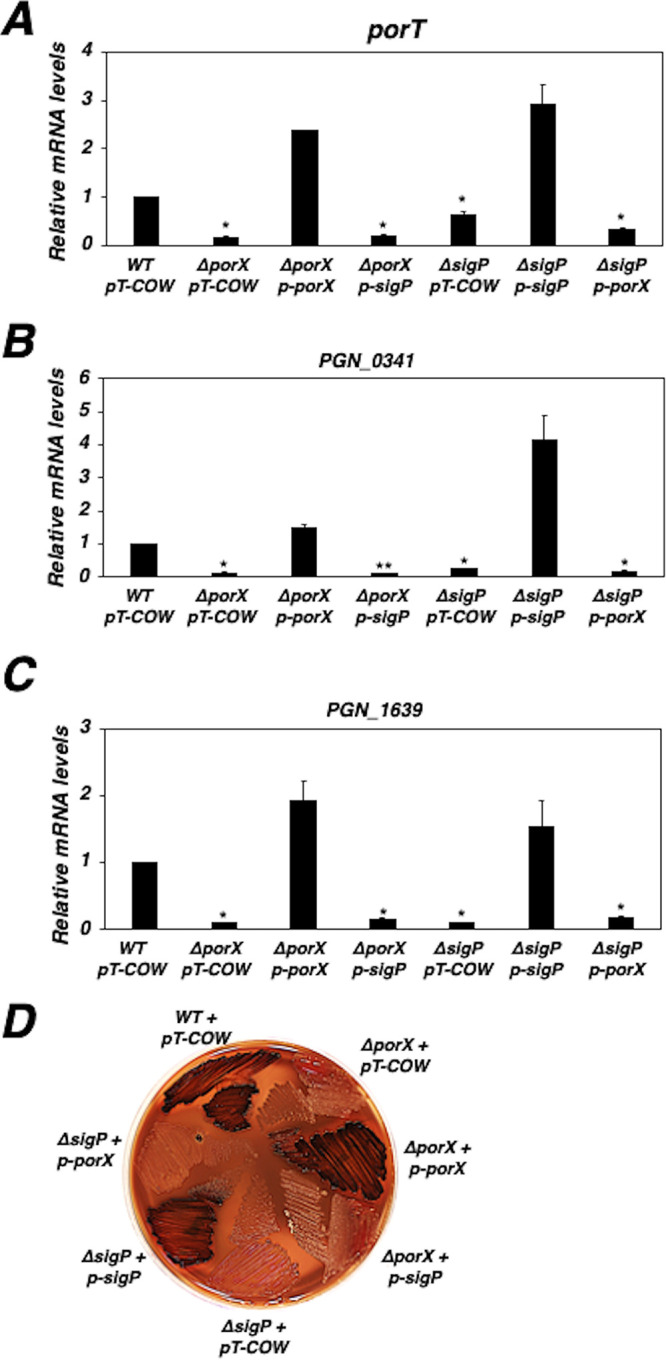
The PorX/PorY system and σ^P^ coordinate transcription in P. gingivalis. (A to C) mRNA levels of the *porT* gene (A), the *PGN_0341* gene (B), and the *PGN_1639* gene (C) in the 33277 wild-type strain, the Δ*porX* mutant (YS19181), and the Δ*sigP* mutant (YS17717) carrying pT-COW, p-*porX* (pYS18679, pT-COW-P*_PGN_1016_*-*porX*), or p-*sigP* (pYS19107, pT-COW-P*_sigP_*-*sigP*). The mRNA level in the wild-type strain was set to 1 for calculation. Results are representative of three independent experiments. *, *P* < 0.05; **, *P* < 0.01; versus wild type by *t* test. (D) The growth of wild-type 33277 strain with pT-COW (vector), Δ*porX* mutant (YS19181), and Δ*sigP* mutant (YS17717) carrying pT-COW, p-*porX*, or p-*sigP*, respectively, on a blood BHI plate containing tetracycline (0.5 μg/ml). Results are representative of four independent experiments.

The PorX/PorY two-component system and extracytoplasmic function sigma factor σ^P^ (encoded by the *sigP* gene) appeared to form a regulatory cascade for upregulation of the T9SS-encoding genes (i.e., the *por* genes) because PorX/PorY was shown to upregulate σ^P^ and, in turn, σ^P^ enhanced the transcription of the *por* genes (5). Particularly, it was observed that the PorX/PorY-stimulated σ^P^ bound the promoters of the *por* genes, including the *porT* gene which encodes a T9SS component (5). Besides σ^P^, a recent result from our laboratory showed the PorX protein also bound to the *porT* promoter and actually interacted with two DNA regions (7). This result not only verified the DNA-binding ability of the PorX response regulator, but also provided the possibility that both PorX and σ^P^ should directly act on the *por* promoters. If PorX and σ^P^ must coordinately control but not build a regulatory cascade to regulate the *por* genes, we postulate that σ^P^ expressed in *trans* in the absence of PorX, or vice versa, should not stimulate *por* transcription. We examined this hypothesis by determining the transcription of the *porT* gene in a *porX* deletion mutant (Δ*porX*). As predicted, a σ^P^ protein that was expressed in *trans* from a plasmid (pT-COW-P*_sigP_*-*sigP*, referred to as p-*sigP*) did not exert any effect on the *porT* expression in the Δ*porX* mutant because the *porT* mRNA level in this mutant harboring p-*sigP* remained similar to that in the mutant harboring the parental plasmid pT-COW (8), both of which were ∼6.4-fold lower than that in the wild-type strain (Fig. 1A). Likewise, PorX had no effect on *porT* expression in the absence of σ^P^, because a PorX protein expressed in *trans* from a plasmid (pT-COW-P*_PGN_1016_*-*porX*, referred to as p-*porX*) did not stimulate *porT* transcription in a *sigP* null (Δ*sigP*) mutant (Fig. 1A). In contrast, the alleviated *porT* transcription was fully reversed to a wild-type level in the Δ*sigP* mutant harboring p-*sigP* and also in the Δ*porX* mutant harboring p-*porX* (Fig. 1A), indicating the *trans*-expressed σ^P^ and PorX proteins were functionally active. We also determined whether this coordinate regulation was effective in controlling two other PorX/PorY- and σ^P^-dependent genes, *PGN_0341*, which encodes a predicted T9SS component (4), and *PGN_1639*, which has been known as a σ^P^-dependent gene (5) and recently identified as a PorX/PorY-dependent locus according to our transcriptomic and proteomic analyses (unpublished data). We confirmed that the transcription levels of *PGN_0341* and *PGN_1639* were upregulated by PorX and σ^P^ because their mRNA levels were significantly reduced in the Δ*porX* and Δ*sigP* mutants compared to those in the wild-type strain (Fig. 1B and 1C). Comparable to the *porT* regulation (Fig. 1A), the alleviated transcription of *PGN_0341* and *PGN_1639* was not stimulated in Δ*porX* mutant harboring p-*sigP* or in Δ*sigP* mutant harboring p-*porX* (Fig. 1B and 1C).

It has been shown that T9SS mediates secretion of gingipains, which are required for pigmentation of P. gingivalis on a blood plate (for review see reference 9), and consistently both Δ*porX* and Δ*sigP* mutants display a nonpigmented phenotype (6, 10). We conducted a phenotypic analysis to evaluate the coordinate interaction between the PorX/PorY system and σ^P^. While the Δ*porX* and Δ*sigP* mutants carrying pT-COW exhibited nonpigmented colonies on a brain heart infusion (BHI) blood plate, both the Δ*porX* mutant harboring p-*porX* and the Δ*sigP* mutant harboring p-*sigP* formed vigorous black-pigmented colonies (Fig. 1D). However, the Δ*porX* mutant harboring p-*sigP* and the Δ*sigP* mutant harboring p-*porX* exhibited a nonpigmented phenotype when they were grown on a BHI blood plate (Fig. 1D). Taken together, these genetic approaches suggest the PorX/PorY system and σ^P^ should govern transcription of the *por* genes via a coherent regulatory network rather than a direct regulatory cascade.

### PorX response regulator directly binds to the promoter of the sigma factor gene *sigP*.

**FIG 2:**
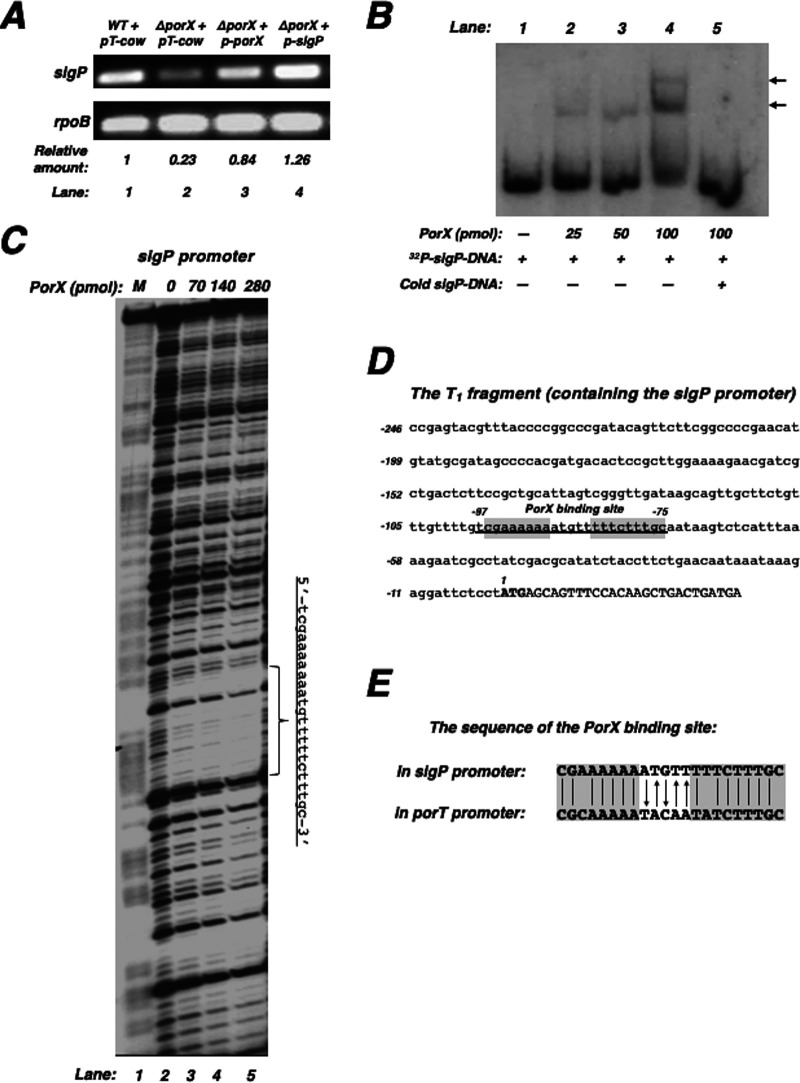
The PorX response regulator binds to the *sigP* promoter region. (A) The mRNA levels of the *sigP* gene in the 33277 wild-type strain and the Δ*porX* mutant (YS19181) carrying pT-COW, p-*porX*, or p-*sigP*. Results are representative of three independent experiments. (B) EMSA analysis for binding of PorX to the *sigP* promoter. ^32^P-labeled *sigP* DNA fragment (40 fmol) was incubated with PorX-_C_-His_6_ protein at the indicated amount. Lane 5 is the same as lane 4 but supplemented with nonlabeled (cold) *sigP* DNA fragment (1 pmol). The PorX/DNA mixtures were subjected to 5% PAGE. The location of DNA migration was detected by autoradiography. Arrows indicate the shifted bands after DNA fragments were associated with the PorX-_C_-His_6_ protein. The experiment was repeated twice. (C) DNase footprinting analysis of the *sigP* promoter fragment amplified with primers ^32^P-3043 and 3044 for the coding strand and increasing amounts of PorX-c-His_6_ protein. Products were separated in polyacrylamide DNA sequencing electrophoresis and the bands were detected by autoradiography. The bracket indicates the region protected by the PorX-_C_-His_6_ protein. Underlined DNA sequence (right of gel) indicates the PorX-protected nucleotides in the *sigP* promoter. The ladder *M* corresponds to the same ^32^P-labeled *sigP* promoter fragment and degraded by the Maxam and Gilbert reaction. Results were repeated multiple times. (D) The DNA sequence of the *sigP* promoter region. Underlining corresponds to the PorX-protected region characterized in (C). Capital letters represent the *sigP* start codon. Numbering begins from the adenine nucleotide of the start codon. Highlighted sequences are shared by the PorX-binding site in the *porT* promoter (also shown in panel E). (E) The homologous sequences of the PorX-binding sites in the *sigP* and *porT* promoters. Vertical lines represent the identical nucleotides in the two sequences. Arrows represent the complementary nucleotides exhibited in the two sequences. Highlighted sequences are shared in these two promoters.

Evidence suggests that transcription of the *sigP* gene is activated by the PorX/PorY system (5). This is confirmed by our result derived from a reverse transcription-PCR, since the *sigP* mRNA level in the Δ*porX* mutant (lane 2, Fig. 2A) was 4.3-fold lower than that in the wild-type strain (lane 1, Fig. 2A). Our result also confirmed the plasmid p-*sigP* should be able to express the *sigP* gene in *trans* because it fully restored the *sigP* mRNA level in the Δ*porX* mutant (lane 4, Fig. 2A). To determine whether the PorX/PorY system can directly upregulate the *sigP* gene, we first characterized the *sigP* promoter region and investigated the PorX binding to this promoter by conducting an electrophoretic mobility shift assay (EMSA) using a 275-bp DNA fragment (marked as T_1_), including the 149-bp intergenic region of the *sigP*-*PGN_0275* genes. We found that a PorX protein with a C-terminal His_6_ tag (referred to as PorX-_C_-His_6_) gel-shifted this DNA fragment in a concentration-dependent manner (Fig. 2B), thus suggesting this T_1_ fragment should contain the *sigP* promoter and also the sequence(s) that binds the PorX protein (i.e., the PorX-binding site). Therefore, we conducted a DNase footprinting assay to map the PorX-binding site in T_1_ and found that the PorX-_C_-His_6_ protein bound to an AT-rich DNA sequence (5′-tcgaaaaaaatgtttttctttgc-3′) in a concentration-dependent manner (Fig. 2C). This PorX-binding site, which is located −97 to −75 nucleotides (nt) upstream of the start codon (underlined nucleotides, Fig. 2D), shared a partial sequence with the PorX-binding site II (5′-gattcgcgcaaaaatacaatatcttt-3′) in the *porT* promoter, recently characterized by our laboratory (7). We postulate that PorX can recognize a sequence (5′-CG(A/C)AAAAA-N_5_-T(T/A)TCTTTGC-3′) that is conserved in these two promoters. Interestingly, the 5 nucleotides located between the conserved segments in the PorX-binding sites of the *sigP* and *porT* promoters were complementary (nucleotides labeled with arrows in Fig. 2E). Therefore, these results and our recent data (7) not only verify that PorX directly regulates transcription of the *sigP* gene and the *por* genes such as *porT*, but also elucidate that PorX is a DNA-binding protein and capable of recognizing specific DNA sequences in a manner similar to many other TCS response regulators.

### PorX protein activates *sigP* transcription *in vitro*.

**FIG 3:**
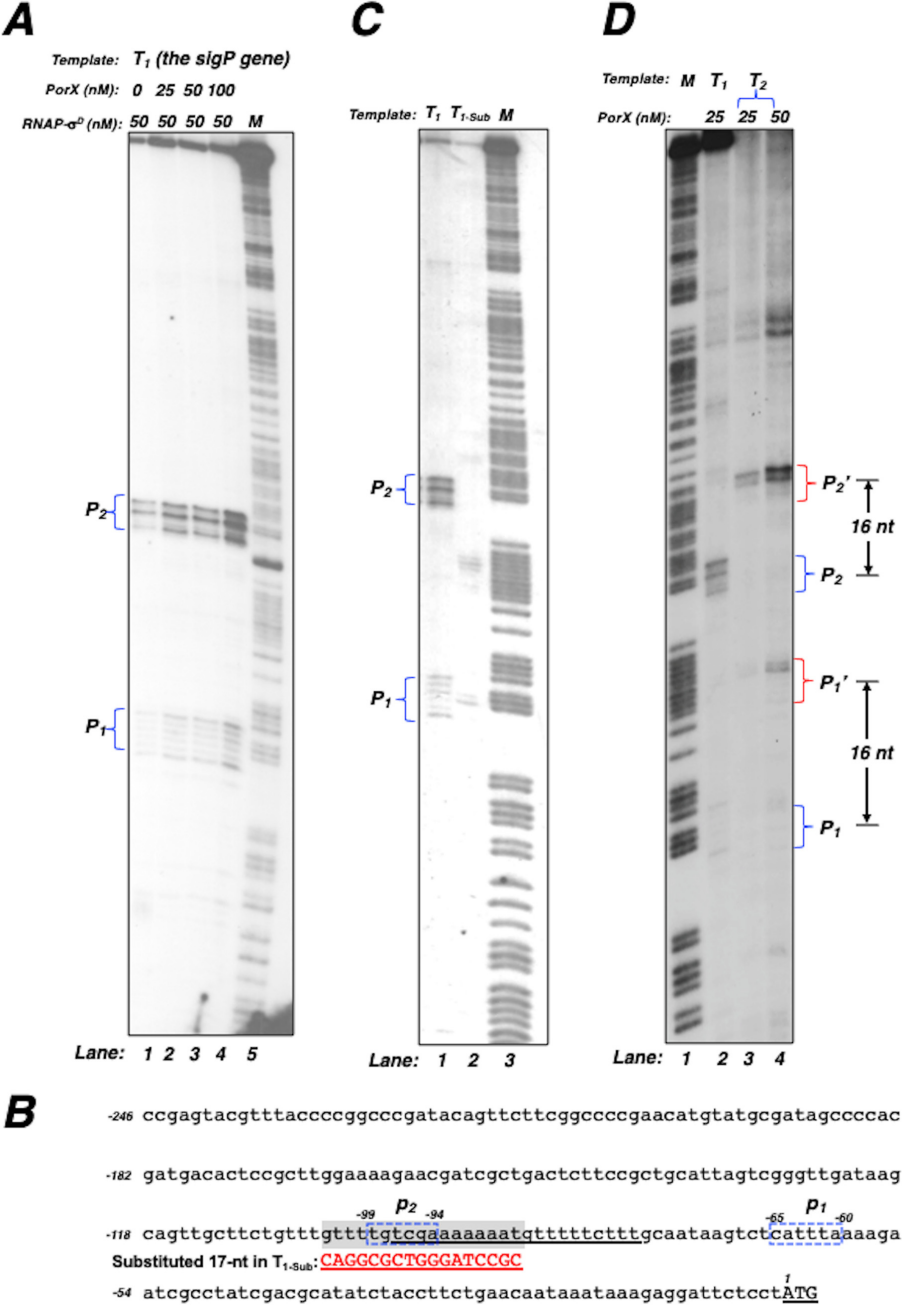
PorX promotes *sigP* transcription *in vitro*, mediated by a reconstituted P. gingivalis RNA polymerase-σ^D^. (A) *In vitro* transcription of a 275-bp template (T_1_) from the *sigP* promoter containing the first 29 coding nucleotides was conducted as described in the Materials and Methods. Left braces indicate the P_1_ and P_2_ transcripts synthesized by 50 nM of RNA polymerase-σ^D^ (RNAP-σ^D^) from reactions supplemented with different amounts of the PorX-_C_-His_6_ protein. The ladder *M* corresponds to a PCR product generated with primers 3044 and ^32^P-labeled primer 3043 and degraded by the Maxam and Gilbert reaction. (B) The DNA sequence of the *sigP* promoter region. Underlining corresponds to the PorX-protected region. Blue dashed frames correspond to the regions labeled as p_1_ and p_2_, respectively, where transcription was initiated. The highlighted sequence corresponds to the wild-type sequence which was substituted by the sequence (Sub) in red capital letters. Numbering begins from the adenine nucleotide of the start codon (underlined capital letters). (C) *In vitro* transcription of the *sigP* templates (T_1_ and T_1-_*_sub_*) with the wild-type sequence and a substituted sequence, respectively. Blue left braces indicate the transcripts, P_1_ and P_2_, produced from the reaction with template T_1_. (D) *In vitro* transcription of the *sigP* templates (T_1_ and T_2_) containing the first 29 and 45 coding nucleotides, respectively. Blue right braces indicate the transcripts, P_1_ and P_2_, produced from the reaction with template T_1_, and red right braces indicate the transcripts P_1_’ and P_2_’, produced from the reaction with template T_2_. Double arrows indicate that P_1_ and P_2_ are 16 nucleotides shorter than P_1_’ and P_2_’, respectively. Results in A, C, and D were repeated two times.

To further validate the direct role of the PorX/PorY system in *sigP* transcription, we conducted an *in vitro* transcription assay using a P. gingivalis RNA polymerase holoenzyme (referred to as pg-RNAP-σ^D^) that was reconstructed from N-terminal His_6_-tagged subunit proteins, including α (PGN_1841), β (PGN_1571), β’ (PGN_1570), and the major sigma factor σ^D^ (PGN_0638) (for details see the Materials and Methods section). When the T_1_ fragment, which was tested for PorX binding (Fig. 2B and C), was used as the template for the *in vitro* transcription reactions supplemented with 50 nM pg-RNAP-σ^D^, two transcripts labeled as P_1_ and P_2_, respectively, were produced (Fig. 3A). Both transcriptions were stimulated by the PorX-c-His_6_ protein because the amount of P_1_ and P_2_ increased in a PorX concentration-dependent manner (lanes 1 to 4, Fig. 3A). These results suggest that *sigP* transcription is initiated from two regions that are located at 65 to 60 nt (labeled as p_1_) and 99 to 94 nt (p_2_) upstream of the start codon, respectively (illustrated in the T_1_ sequence, Fig. 3B). To verify whether these transcripts were produced specifically, we compared the *in vitro* transcripts from the wild-type T_1_ template and a mutated T_1_ template (T_1-Sub_) which carried 17-nt substitutions at a 103- to 87-nt sequence located upstream of the start codon. Our results showed that levels of both P_1_ and P_2_ transcripts from a reaction using the T_1-Sub_ template were much lower than those using the T_1_ template (lane 2 versus lane 1, Fig. 3C). Since this substituted sequence overlaps a partial region of the PorX-binding site for the P_1_ transcription and the p_2_ region (Fig. 3B), we reasoned that these substitutions must simultaneously interfere with transcription initiated from p_1_ and p_2_ in T_1-Sub_. To further verify that the transcription initiation from p_1_ and p_2_ was specific, we used another template, i.e., T_2_, which was a longer template (291 bp) and contained an additional 16-bp sequence extending from downstream of the T_1_ template (275-bp). The *in vitro* transcription using this T_2_ template could still produce two transcripts, labeled as P_1_’ and P_2_’, in a PorX concentration-dependent manner (lanes 3 and 4, Fig. 3D), and both products were exactly 16-nt longer than P_1_ and P_2_, respectively (lanes 3 and 4 versus lane 2, Fig. 3D). Therefore, the *in vitro* transcription of the *sigP* gene must be specifically initiated from two DNA regions, p_1_ and p_2_, thus allowing the T_1_ template to produce P_1_ and P_2_ transcripts and also the 16-bp longer T_2_ template to produce 16-nt longer P1’ and P_2_’ transcripts.

### PorX stimulates *in vitro* transcription of the *porT* gene carried out by a reconstructed RNA polymerase-σ^P^ holoenzyme.

**FIG 4:**
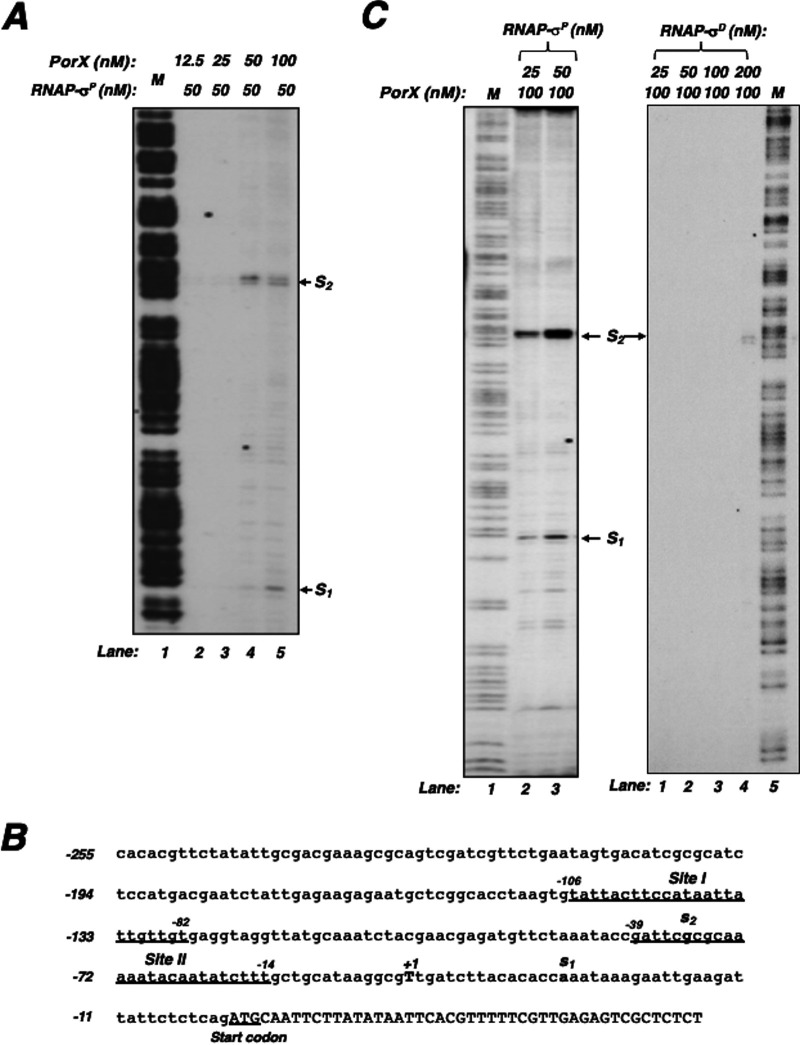
PorX and σ^P^ promote *porT* transcription *in vitro.* (A) *In vitro* transcription of a *porT* template containing its promoter and the first 48 coding nucleotides was conducted as described in the Materials and Methods. The left panel represents the transcripts, labeled as S_1_ and S_2_, respectively, synthesized in the reactions with different amounts of RNAP-σ^P^ with and 100 nM PorX-_C_-His_6_ protein. The right panel represents the products synthesized in the reactions with different amounts of RNAP-σ^D^ and 100 nM PorX-c-His_6_ protein. The ladder *M* corresponds to a PCR product generated with primers 4026 and ^32^P-labeled primer 4025 and degraded by the Maxam and Gilbert reaction. (B) The DNA sequence of the *porT* promoter region. Underlined sequences correspond to the PorX-protected regions and are also labeled as I and II, respectively. Bold letters, labeled as s_1_ and s_2_, correspond to the transcription initiation sites detected from the *in vitro* transcription. Underlined capital letters present the *porT* start codon. (C) *In vitro* transcription of *porT* in the reactions supplemented with 50 nM RNAP-σ^P^ and different amounts of PorX-_C_-His_6_ protein. The ladder *M* is the same as in A. Results in A and C were repeated two times.

Since PorX directly binds to the σ^P^-dependent *porT* promoter (7), we postulated that it should be able to stimulate *porT* transcription *in vitro*. To examine this hypothesis, we conducted an *in vitro* transcription assay using a P. gingivalis RNA polymerase σ^P^ holoenzyme (referred to as pg-RNAP-σ^P^) which was reconstructed from purified N-terminal His_6_-tagged α, β, β’ and C-terminal His_6_-tagged σ^P^ proteins (for details see the Materials and Methods section). When a 301-bp DNA fragment, including the *porT* promoter sequence, was used as the template, two transcripts labeled as S_1_ and S_2_ were produced by the reconstructed pg-RNAP-σ^P^ (at 50 nM) and both transcriptions were enhanced by PorX in a concentration-dependent manner (lanes 2 to 5, Fig. 4A). S_1_ transcription was initiated from the adenosine (labeled as s_1_, Fig. 4B) located 29 nucleotides downstream of PorX binding site II, identified in our previous study (7). S_2_ transcription was initiated from the guanosine (labeled as s_2_, Fig. 4B) located 49 nucleotides downstream of PorX binding site I in the *porT* promoter. Thus, we postulated that PorX should bind to site I and site II and enhance the transcription initiated at s_2_ and s_1_, respectively. Synthesis of both S_1_ and S_2_ was significantly stimulated when the pg-RNAP-σ^P^ concentration was elevated from 25 nM to 50 nM (lanes 2 and 3, left panel, Fig. 4C). In contrast, the pg-RNAP-σ^D^ holoenzyme was not as efficient as pg-RNAP-σ^P^ because only S_2_ could be produced to a detectable level by pg-RNAP-σ^D^ at a high concentration of 200 nM (lane 4, right panel, Fig. 4C). These observations suggest that σ^P^ should be the preferred sigma factor to mediate the *porT* transcription and that both PorX and σ^P^ act directly on its promoter. Interestingly, the s_1_ and s_2_ sites did not overlap the transcription initiation site (+1) detected from a primer extension assay using a total wild-type mRNA sample (7). This is probably because other factors in the bacterial cell might interact with PorX and RNA polymerase-σ^P^ holoenzyme to initiate the *porT* transcription from the +1 position.

### PorX/PorY system is essential for the virulence of P. gingivalis in a mouse model.

**FIG 5:**
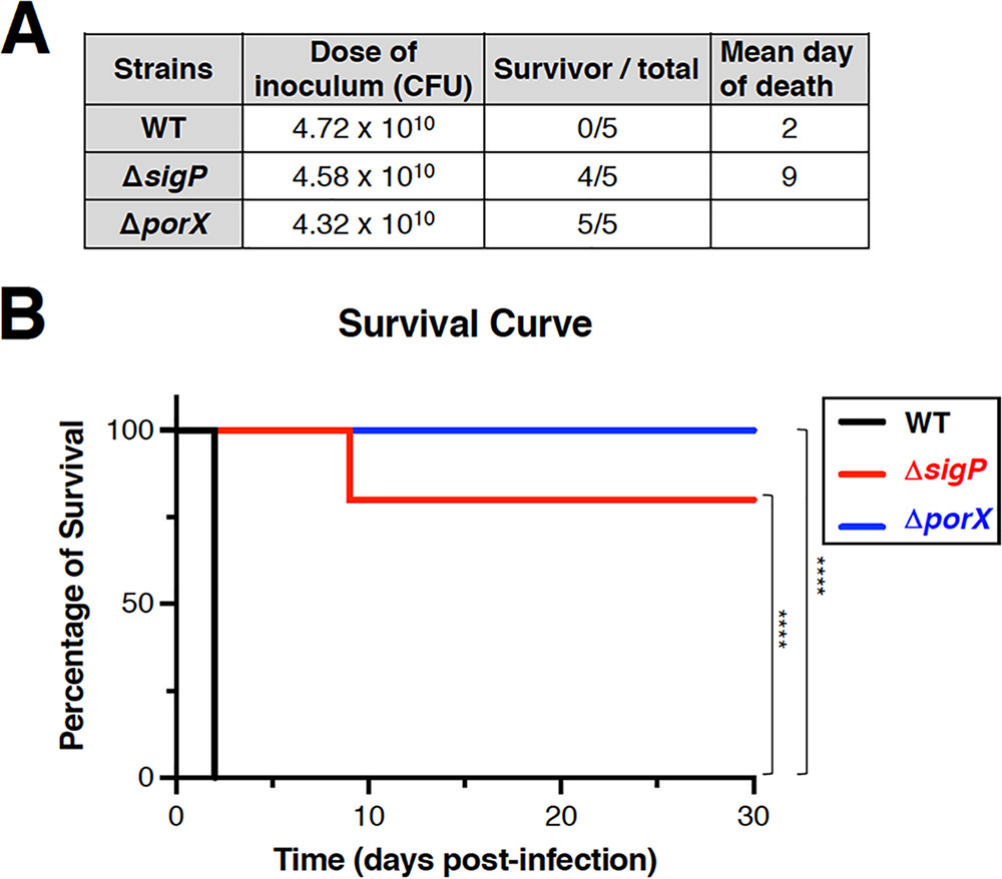
The PorX/PorY-determined virulence of P. gingivalis W83 strains. (A) Virulence test using groups of BALB/c mice (*n *= 5) that were subcutaneously injected with P. gingivalis W83 wild-type, Δ*sigP* (YS18145), and Δ*porX* (YS19145) strains, respectively. (B) Survival curves of the results from A (*n *= 5; *P* < 0.0001). Three sets of experiments were carried out.

According to our previous results (7), the PorX/PorY system is a virulence regulator of P. gingivalis because a virulent W83 wild-type strain, but not the Δ*porX* mutant, could cause infection in a mouse model described previously (11). To determine whether the PorX/PorY-activated σ^P^ contributes to bacterial virulence, we compared the pathogenesis of this wild-type strain and its isogenic Δ*sigP* mutant in this mouse model. Six-week-old BALB/c mice were subcutaneously injected on the dorsal surface with the strains that were grown in BHI medium for 12 h, and all five mice that were challenged by W83 wild-type cells at a dose of 4.72 × 10^10^ CFU died in 48 h (Fig. 5A and 5B). On the other hand, four out of the five mice challenged with the isogenic Δ*sigP* mutant cells at a dose of 4.58 × 10^10^ CFU survived the 30-day observation period (Fig. 5A and 5B), thus demonstrating that the sigma factor σ^P^ is a virulence determinant. The Δ*sigP* mutant was highly attenuated but not as avirulent as the Δ*porX* mutant, which, at a dose of 4.32 × 10^10^ CFU, did not kill even one mouse in the 30-day observation period (Fig. 5A and 5B). The result of the Δ*porX* mutant also reconfirmed that the PorX/PorY system is essential for P. gingivalis virulence (7). Accordingly, we postulated that the PorX/PorY system should also be able to activate other P. gingivalis virulence factors whose regulation is independent of σ^P^. Based on these observations, it is reasonable to assume that the PorX/PorY system renders P. gingivalis virulent in part by activating the *sigP* gene in this mouse model. This assumption should be further confirmed by our ongoing RNA sequencing analysis, which compares the expression of overall PorX/PorY- and σ^P^-regulated genes in P. gingivalis cells recovered from the animal against those grown *in vitro*.

In conclusion, pathogenic bacteria have developed many sophisticated mechanisms to control the expression of the genes that contribute to virulence. Growing evidence suggests that the PorX/PorY system in P. gingivalis plays an essential role in the regulation of numerous virulence determinants, exemplified by the set of *por* genes encoding the T9SS components. This study has revealed that the PorX/PorY system and sigma factor σ^P^ construct a regulatory pathway to coordinate the regulation of the *por* genes. We provide evidence that the PorX response regulator binds to the *sigP* promoter (Fig. 2B and 2C) and activates the *sigP* transcription in an *in vitro* transcription reaction system using a reconstructed RNA polymerase holoenzyme (Fig. 3A, C, and D), thus demonstrating that the PorX/PorY system directly regulates transcription of the *sigP* gene.

**FIG 6:**
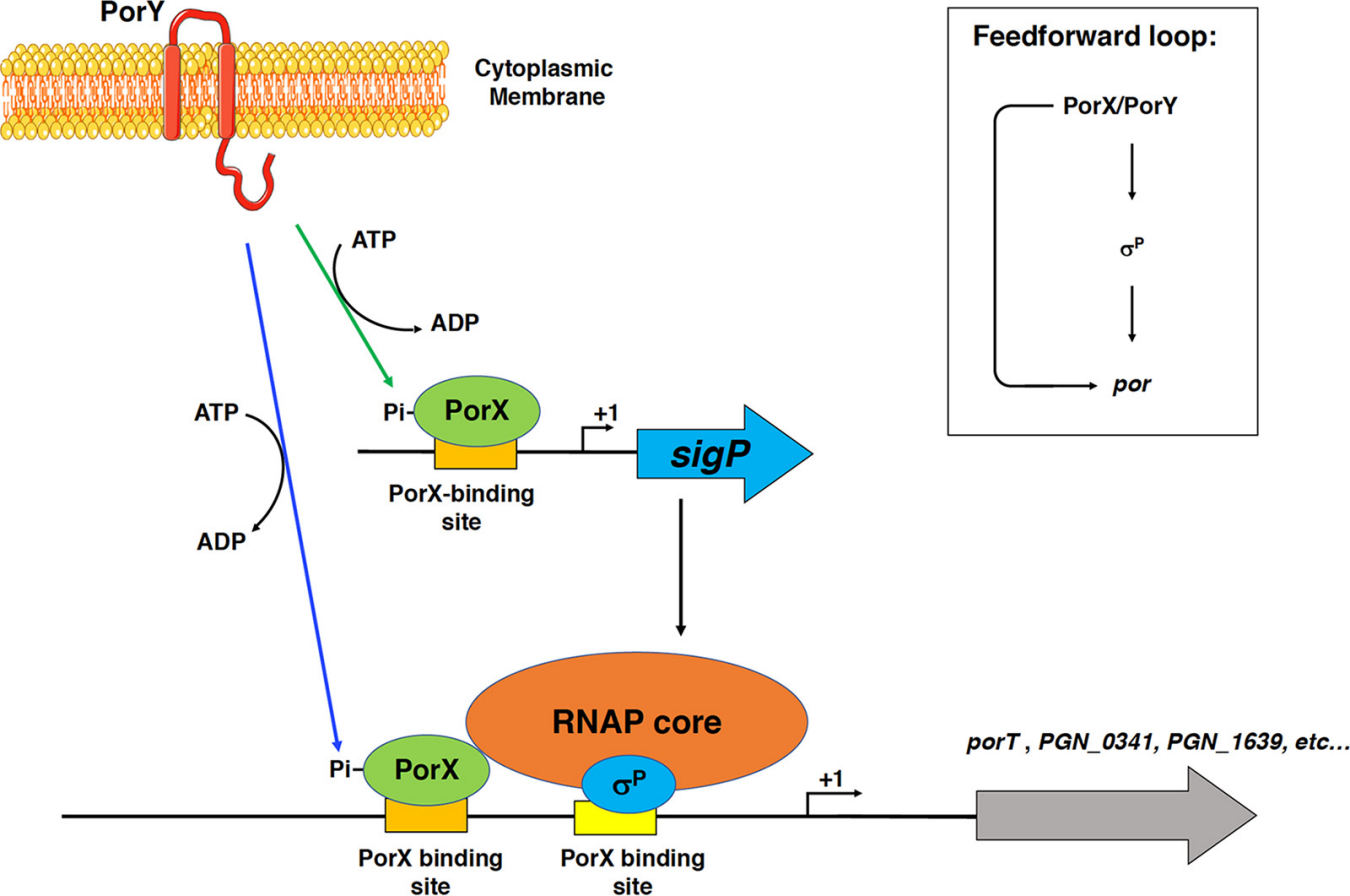
Feedforward loop model illustrating the PorX/PorY- and σ^P^-dependent regulatory mechanism. In P. gingivalis, PorX/PorY and σ^P^ build a feedforward loop. The PorY sensor kinase phosphorylates its cognate PorX response regulator. The phosphorylated PorX protein binds to the *sigP* promoter at the PorX-binding site and upregulates transcription of the *sigP* gene. The PorX/PorY-stimulated σ^P^ protein and RNA polymerase core enzyme build a holoenzyme. Then, phosphorylated PorX protein and RNAP σ^P^ holoenzyme coordinately activate transcription of their target genes by simultaneously binding to their promoters at the PorX-binding sites and the σ^P^ recognition site, respectively. The inset illustrates the PorX/PorY σ^P^ feedforward loop that modulates *por* expression.

When two related regulators build a regulatory cascade, the first regulator regulates the second regulator, and then the second regulator regulates their target genes. Therefore, in the absence of the first regulator, the target genes will still be regulated by the second regulator when this regulator can be produced in *trans*. Although PorX/PorY activates σ^P^, and then σ^P^ activates the *por* genes, this regulatory cascade model is inapplicable to the regulation dependent on the PorX/PorY system and σ^P^ because both the first regulator (PorX) and the second regulator (σ^P^) are shown to bind to the *por* promoters (5, 7), and σ^P^ produced in *trans* from p-*sigP* is unable to activate these genes in the Δ*porX* mutant (Fig. 1A to C). We also show that the PorX protein can directly enhance *in vitro porT* transcription catalyzed by an RNA polymerase-σ^P^ holoenzyme (Fig. 4A and 4C), which further confirms the direct action of PorX on the *porT* promoter. Therefore, regulation of the *por* genes should be controlled coordinately by the PorX/PorY system and σ^P^, in which PorX stimulates the production of σ^P^, and both PorX and σ^P^ regulate the *porT* transcription. We postulate that this mechanism of action of the PorX/PorY system and σ^P^ should fall under the criteria of a regulatory motif, which is known as the feedforward loop (12) (Fig. 6). Our previous results have shown that the PorX/PorY system responds to hemin and enhances transcription of the *porT* gene (7). In many cases, the feedforward loop has the capability to integrate multiple signaling molecules into a gene regulation (12). It remains to be investigated whether the feedforward loop contributing to the PorX/PorY- and σ^P^-governed signal transduction pathway is able to respond to signal molecules besides hemin.

It is worth noting that the transcription of the *sigP* gene in the Δ*porX* mutant is not completely repressed (5) (Fig. 2A). According to a previous study (13), σ^P^ exerts an inhibitory effect on P. gingivalis biofilm formation, as biofilm formation is induced in a *sigP* null mutant in an enriched BHI medium. However, the Δ*porX* mutant grown in this BHI medium did not induce biofilm formation (unpublished result). We reason that the expression of the *sigP* gene remaining in the Δ*porX* mutant is sufficient to inhibit biofilm formation.

The PorX/PorY system has been shown as an essential regulator for P. gingivalis virulence since the Δ*porX* mutant is avirulent in mouse infection (7) (Fig. 5A and B). In this study, the murine virulence assay has demonstrated that σ^P^ contributes to P. gingivalis virulence and the Δ*sigP* mutant becomes attenuated. Further *in vivo* analysis will be needed to confirm the role of σ^P^ in the PorX/PorY-controlled mechanism required for P. gingivalis pathogenesis.

## MATERIALS AND METHODS

### Bacterial strains, plasmids, media, and growth conditions.

Strains and plasmids used in this study are listed in Table 1. The P. gingivalis ATCC 33277 and W83 wild-type strains used in this study were obtained from Koji Nakayama (4). P. gingivalis cells were grown at 37°C in an anaerobic chamber (Model 2000, Coy Lab Products) that maintained 90% N_2_/5% CO_2_/5% H_2_ in the atmosphere. Blood agar plates (5% sheep defibrinated blood, 1.5% agar) or brain heart infusion (BHI, purchased from BD) medium supplemented with hemin (5 μg/ml) were used to culture P. gingivalis strains. When necessary, erythromycin (0.5 μg/ml) or tetracycline (0.5 μg/ml) was supplemented. P. gingivalis cells were harvested by centrifuging liquid cultures at 10,000 × *g* (∼8,500 rpm) in a Sorvall ST 8R centrifuge with a HIGHConic III fixed angle rotor (maximum 9,500 rpm) at 4°C for 10 min. E. coli DH5*α* and BL21(DE3) strains were used for cloning and protein production, respectively. E. coli cells were routinely grown in Luria broth (LB) supplemented with antibiotics when necessary (kanamycin, 50 μg/ml; ampicillin, 50 μg/ml) at 37°C. To prepare cell lysates, bacterial cells were opened with a sonicator (Misonix Sonicator 3000).

**TABLE 1:** Bacterial strains and plasmids used in this study

Strain or plasmid	Description	Reference or source
Porphyromonas gingivalis		
ATCC 33277	Wild type	
YS19181	Δ*porX*::Em^R^	(7)
YS17717	Δ*sigP*::Em^R^	This work
W83	Wild type	
YS19145	Δ*porX*::Em^R^	(7)
YS18145	Δ*sigP*::Em^R^	This work
E. coli		
DH5*α*	F^−^ *sup*E44 Δ*lac*U169 (ϕ80 *lacZ* Δ*M15*) *hsdR*17 *recA*1 *endA*1 *gyrA*96 *thi*-1 *relA*1	Lab collection
		(21)
BL21(DE3)	F^−^ *ompT hsdS*_B_ (r_B_^−^ m_B_^−^ ) *gal dcm* (DE3)	(22)
Plasmids		
pT-COW	rep_ColE1_ Amp^R^ Tc^r^	(14)
pYS18679	rep_ColE1_ rep_pB8-51_ Amp^R^ Cam^R^ Tc^R^ P_PGN_1016_ *porX*_CDS_	(7)
pYS19107	rep_ColE1_ rep_pB8-51_ Amp^R^ Cam^R^ Tc^R^	This work
	*sigP*_CDS_	This work
pET28a	rep_ColE1_ Km^R^ *lacI* P_T7_	Novagen
pET11a	rep_ColE1_ Amp^R^ *lacI* P_T7_	Novagen
pET21a	rep_ColE1_ Amp^R^ *lacI* P_T7_	Novagen
pGEM-T-Easy	rep_pMB1_, _f1_ Amp^R^ *lacZ-α*	Promega
pGEM-*ermF*	rep_ColE1_ Amp^R^ *ermF lacI* P_T7_	(16)
pYS17676	rep_ColE1_ Amp^R^ *ermF lacI* P_T7_ *sigP*_8-305nt_	This work
pYS18456	rep_ColE1_ Amp^R^ *lacI* P_T7_ *porX* (*PGN_1019*)	(7)
pYS18051	rep_ColE1_ Km^R^ *lacI* P_T7_ *rpoA* (*PGN_1841*)	This work
pYS18943	rep_ColE1_ Amp^R^ *lacI* P_T7_ *rpoB* (*PGN_1571*)	This work
pYS18165	rep_ColE1_ Km^R^ *lacI* P_T7_ *rpoC* (*PGN_1570*)	This work
pYS18052	rep_ColE1_ Km^R^ *lacI* P_T7_ *rpoD* (*PGN_0638*)	This work
pYS18056	rep_ColE1_ Km^R^ *lacI* P_T7_ *sigP* (*PGN_0274*)	This work

### Construction of plasmids and strains with chromosomal mutations.

All plasmids used in this study are listed in Table 1. Polymerase chain reactions (PCR) were performed using a Bio-Rad T100 thermal cycler with *Taq* DNA polymerase (New England BioLabs [NEB]). Custom oligonucleotides were synthesized by Integrated DNA Technologies (IDT) and are listed in Table 2. PCR products were isolated using a QIAquick PCR purification kit (Qiagen). Restriction enzymes were purchased from New England BioLabs and used according to the manufacturer’s instructions. Digested DNA fragments were separated in 0.8 to 1% agarose gels and then isolated using a QIAquick gel extraction kit (Qiagen). Plasmids were purified from overnight cultures of E. coli DH5α in LB at 37°C using plasmid minikit or midi kit (Qiagen). Plasmid pYS19107 for complementation assays was constructed using PCR fragments containing a 500-bp sequence of the upstream region followed by the *sigP* coding region, which was amplified with primers 3809 and 2827, digested with HindIII and BamHI, and ligated between the HindIII and BamHI sites of pT-COW (14). Plasmid pYS17676 for mutagenizing the *sigP* gene in both 33277 and W83 strains was constructed using a DNA fragment containing the 8- to 305-nt *sigP* coding region amplified with primers 2768 and 2769, digested with PstI, and then ligated with PstI-digested pGEM-*ermF* plasmid. Plasmid pYS18051 was constructed using PCR fragments containing the *rpoA* (*PGN_1841*) coding region amplified with primers 3158 and 3159, digested with NcoI and BamHI, and then ligated between the NcoI and BamHI sites of plasmid pET28a. Plasmid pYS18943 was constructed using PCR fragments containing the *rpoB* (*PGN_1571*) coding region amplified with primers 3160 and 3161, digested with NheI, and then ligated between the NheI sites of plasmid pET11a. Plasmid pYS18165 was constructed using PCR fragments containing the *rpoC* (*PGN_1570*) coding region amplified with primers 3162 and 3163, digested with NcoI and XhoI, and then ligated between the NcoI and XhoI sites of plasmid pET28a. Plasmid pYS18052 was constructed using PCR fragments containing the *rpoD* (*PGN_0638*) coding region amplified with primers 3164 and 3165, digested with NcoI and HindIII, and then ligated between the NcoI and HindIII sites of plasmid pET28a. Plasmid pYS18056 was constructed using PCR fragments containing the *sigP* coding region amplified with primers 3148 and 3149, digested with NcoI and XhoI, and then ligated between the NcoI and XhoI sites of plasmid pET28a. All plasmids were sequenced before use. The P. gingivalis Δ*sigP* mutant was constructed by introducing suicide plasmid pYS17676 into the 33277 and W83 wild-type strains, respectively, using an electroporation procedure described previously (4). Mutated sequences in these strains were confirmed by DNA sequencing.

**TABLE 2:** Primers used in this study^*a*^

Primer no.	Sequence
2499	gga aga gaa gac cgt agc aca agg a
2500	gag tag gcg aaa cgt cca tca ggt c
2741	ccc aag ctt gac aca gca gca gga aaa gc
2742	cgc gga tcc tta ctt ggg ttg cat cgt aat
2768	aaa act gca ggt ttc cac aag ctg act g
2769	aaa act gca gtg caa cct ggc tcc ttc c
2827	ccg ctc gag cta agc cga cat gcc cat c
3043	tca tca gtc agc ttg tgg
3044	ccg agt acg ttt acc cc
3148	cat gcc atg gca atg agc agt ttc cac aag c
3149	ccg ctc gag agc cga cat gcc cat
3158	cat gcc atg ggc cat cat cat cat cat cac gca ata tta gca ttt cag
3159	cgg gat cct tat tag tct tta tct aat tta tac
3160	cta gct agc cat cat cat cat cat cac acg ccg act aca aac aac
3161	cta gct agc tta tta gtc caa aga aaa act t
3162	cat gcc atg ggc cat cat cat cat cat cac gct ttt aga aaa gaa aat aag
3163	ccg ctc gag tta cta ttc cga tgg tgc ttc
3164	cat gcc atg ggc cat cat cat cat cat cac agg caa ctt aaa att tcc
3165	ccc aag ctt tta tta gcc gag ata acc ttt cag
3264	cgg tcg gag gca gga atg
3470	gtt cgt tcg cga ata tgc
3471	cga gga cag tag ctt tgg
3764	cca aag cta ctg tcc tcg
3765	tac gaa ggc atc gaa agg
3809	cgc gga tcc caa cta ctg cta ctg tct c
3837	atg tag gga tgc atg ccc
3838	caa agt cgg aag caa acg
3912	gtc agt tct tcc act cgg
3913	gga aga atg gtc aga tcg
4025	aga gag cga ctc tca acg
4026	cac acg ttc tat att gcg
4105	cag gcg ctg gga tcc gcg ttt ttc ttt gca ata ag
4106	cgc gga tcc cag cgc ctg aaa cag aag caa c
4111	aag gct gac caa ttc atc
4193	cgg ccg aat gcg ata tgc
4194	agc ata ttc gcc aaa agg

a All oligonucleotides were purchased from IDT (Integrated DNA Technologies)

### Quantitative real-time PCR.

Bacterial cells were grown anaerobically in BHI medium at 37°C for 48 h. Total RNA was isolated from bacterial cultures using a High Pure RNA isolation kit (Roche) according to the manufacturer’s instructions. The concentration of RNA samples was determined by measuring absorbance at 260 nm using a spectrophotometer (SmartSpec Plus, BIO-RAD). The quality of RNA was evaluated in a 1.2% agarose gel. cDNAs were synthesized using random primers (IDT) and a murine leukemia virus reverse transcriptase (NEB). The amount of cDNA was quantified using PowerUp SYBR green Master Mix according to the manufacturer’s instructions with primers 3837 and 3838 for *porT*, 3912 and 3913 for *PGN_0341*, 3764 and 3765 for *PGN_1639*, 4193 and 4194 for *sigP*, and 2499 and 2500 for *rpoB* (Table 2) and qPCR was performed in QuantStudio 3 Real-time PCR systems (Applied Biosystems, Thermo Fisher Scientific).

### Isolation of the RpoA-_N_-His_6_, RpoB-_N_-His_6_, RpoC-_N_-His_6_, σ^D^-_N_-His_6_, σ^P^-_C_-His_6_, and PorX-_C_-His_6_ proteins.

E. coli BL21-Gold (DE3) harboring plasmids pYS18051, pYS18943, pYS18165, pYS18052, pYS18056, and pYS18456, respectively, were grown in 500 ml of LB medium by shaking at 37°C to an optical density at 600 nm (OD_600_) value of 0.5, then IPTG (isopropyl-β-D-thiogalactopyranoside) was added to a final concentration of 0.4 mM, and bacterial cells were cultured for another 2 h. Bacterial cells were harvested by centrifuge at 10,000 × *g* for 15 min and washed with 50 ml of phosphate-buffered saline (PBS) once, suspended in 10 ml of PBS, and opened by sonication (Misonix Sonicator 3000). The cell lysate was used for purification of the RpoA-_N_-His_6_, RpoB-_N_-His_6_, RpoC-_N_-His_6_, σ^D^-_N_-His_6_, σ^P^-c-His_6_, and PorX-c-His_6_ proteins with Ni-NTA Affinity Gel (Qiagen) by following the manufacturer’s instructions. The purity and concentration of protein samples were determined using a Silver Staining kit (Pierce) and BCA Protein assay kit (Pierce) by following the instructions from the manufacturer.

### Electrophoretic mobility shift assay.

The electrophoretic mobility shift assay (EMSA) was performed as described (15) with the following modifications. Primer 3043 was labeled using T4 polynucleotide kinase (New England BioLabs) and [γ-^32^P]ATP (PerkinElmer Life Sciences). Ten nanomoles of ^32^P-labeled DNA fragments containing the 275-bp *sigP* promoter region, amplified by PCR from 33277 chromosomes with primers 3044 and ^32^P-labeled 3043, were incubated at room temperature for 30 min with 0, 25, 50, or 100 pmol of PorX-_C_-His_6_ protein in 20 μl of an EMSA buffer consisting of 10 mM Tris-HCl (pH 7.5), 1 mM EDTA, 5 mM dithiothreitol (DTT), 10 mM NaCl, 1 mM MgCl_2_, and 5% glycerol. After the addition of the DNA dye solution (40% glycerol, 0.05% bromophenol blue, 0.05% xylene cyanol), the mixture was directly subjected to 4% polyacrylamide electrophoresis. Signals were detected by autoradiography.

### DNase footprinting analysis.

The DNase I footprinting assay was performed as described (15) with the following modifications. ^32^P-labeled DNA (25 pmol, as was used for EMSA) was mixed with 0, 70, 140, or 280 pmol of the PorX-_C_-His_6_ protein in a 100 μl reaction. DNase I digestion was carried out using 0.05 units DNase I (Invitrogen) per reaction. Samples were analyzed by 6% denaturing polyacrylamide electrophoresis by comparison with a DNA sequence ladder generated by Maxam and Gilbert A+G reaction, using the same ^32^P-labeled PCR product. The positions of radioactive DNA fragments in the gels were detected by autoradiography.

### Reconstitution of RNAP holoenzymes from isolated subunits.

A procedure for reconstitution of E. coli RNAP holoenzyme developed and described in detail (16) was successfully used for reconstitution of other bacterial RNAPs. We used a modified procedure presented in a previous study (17) to carry out reconstitution of P. gingivalis RNAP holoenzymes with the following modifications. Briefly, prior to the *in vitro* reconstitution, RNAP subunits isolated from the procedure above were suspended in a denaturation buffer (6 M guanidine-HCl, 50 mM Tris-HCl [pH 7.9], 10 mM MgCl_2_, 10 μM ZnCl_2_, 10% glycerol, 1 mM EDTA, and 10 mM DTT). The mixtures were left for 30 min on ice and then spun in a 4°C microcentrifuge at 10,000 × *g* for 30 min. The supernatants were transferred into fresh tubes and the protein concentration was determined using the BCA protein assay kit (Pierce) with bovine serum albumin (BSA) as a standard. RNAP subunits were mixed in a molar ratio of 2:8:4 (a:β:β′) and dialyzed against 250-volume reconstitution buffer (50 mM Tris-HCl [pH 7.9], 200 mM KCl, 10 mM MgCl_2_, 10 μM ZnCl_2_, 10% glycerol, 1 mM EDTA, and 10 mM 2-mecaptoethonal) at 4°C for 16 h with two changes. One molar equivalent of isolated RNAP σ subunit (σ^D^ or σ^P^) in PBS was added to the supernatant and the mixture was incubated at 30°C for 1 h. The resulting RNAP preparations were used directly in transcription assays or stored under (NH_4_)_2_SO_4_ (65% saturation) until further use.

### Transcription of *sigP* and *porT in vitro*.

The *in vitro* transcription was conducted in a 50-μl reaction mixture containing 1× *in vitro* transcription buffer (80 mM HEPES-KOH [pH 7.5], 24 mM MgCl_2,_ 2 mM spermidine, 40 mM DTT with 500 μM ATP, CTP, GTP, and UTP, respectively) and 1 μg of linear double-stranded DNA (dsDNA) template with the desired amounts of PorX-c-His_6_ protein and an RNA polymerase holoenzyme. Reaction mixtures were incubated for 2 h at 37°C and transcripts were precipitated using three volumes of cold 100% ethanol and 1/10 volume of 3 M sodium acetate (pH 5.8) and resuspended with RNase-free water. For *sigP* transcription *in vitro*, the template T_1_ was amplified from 33277 chromosomal DNA using primers 3043 and 3044, while T_1-_*_Sub_* with substituted PorX binding sequence (from gttttgtcgaaaaaaat to caggcgctgggatccgc) was prepared with primers 3043 and 3044 and 4105 and 4106 by using an overlap extension PCR (18). The longer template T_2_ was amplified from 33277 chromosomal DNA using primers 3044 and 4111. For *porT* transcription *in vitro*, the template was amplified from 33277 chromosomal DNA using primers 4025 and 4026. Transcripts *in vitro* were monitored after being converted into cDNAs through a primer extension performed as described (19) with the following modifications. RNA pellets derived from templates T_1_ and T_1-_*_Sub_* were reverse transcribed using 2 μl of ^32^P-labeled primer 3043 in a 20-μl mixture containing 25 units of M-MuLV reverse transcriptase (NEB) at 42°C for 2 h. ^32^P-labeled primer 4111 was used for primer extension of the transcripts derived from template T_2_. Transcripts derived from the *porT* template were reverse transcribed by using ^32^P-labeled primer 4025. The cDNA samples were precipitated with 2.5 volumes of ethanol and 0.3 M sodium acetate (pH 5.8) and resuspended in 5 μl of Gel Loading Buffer II (Thermo Fisher), and then analyzed by a 6% denaturing polyacrylamide gel. DNA ladders were amplified from 33277 chromosomal DNA using three primer pairs (^32^P-labeled 3043 and 3044; ^32^P-labeled-4111 and 3044; and ^32^P-labeled-4025 and 4026) for the products from T_1_, T_2_, and the *porT* template, respectively, and generated by Maxam-Gilbert reaction.

### Virulence assay in a mouse model.

All animal experiments conform to our animal protocols (18‐1655R) approved by the Institutional Animal Care and Use Committee (IACUC), Office of Research Integrity and Assurance, Arizona State University (ASU protocol number 18‐1655R). Groups of 6-week-old female BALB/c mice (purchased from Charles River Laboratories) were randomly allocated into different groups. Determination of virulence of the P. gingivalis W83 and mutant strains was performed using mouse subcutaneous infection experiments, as described previously (20), with slight modifications. Briefly, bacterial cells were grown in enriched BHI broth at 37°C for 12 h. The culture was diluted 20-fold in 100 ml of fresh BHI medium and grown for the time periods indicated. The cells were harvested by centrifugation at 10,000 × *g* for 20 min and washed once with PBS, then adjusted to a concentration of approximately 5 × 10^11^ CFU/ml in PBS. Resulting bacterial cultures were serially diluted and plated for bacterial CFU to determine the exact titer of all strains used for infections. Mice were challenged with subcutaneous injections of 0.1 ml at each of the two sites on the depilated dorsal surface (0.2 ml per mouse). Infected mice were examined daily for survival.

### Statistics.

Each *in vitro* experiment was conducted at least three times independently. Mice were randomly placed into different groups before tests. A Kaplan Meier curve was used for survival analysis in this study. Comparisons between two groups were performed with Student’s *t* test and *P ≤ *0.05 was considered significant. Statistics were calculated with GraphPad Prism version 8.0.
